# The risks and benefits of RTPA in acute ischemic stroke for patients at high risk of intracranial haemorrhage and poor functional outcome: a secondary analysis of the IST-3 trial and systematic review of prediction models

**DOI:** 10.1186/1745-6215-14-S1-O9

**Published:** 2013-11-29

**Authors:** Douglas Thompson, Gordon Murray, William Whiteley

**Affiliations:** 1 Edinburgh MRC Hub for Trials Methodology Research, University of Edinburgh, Edinburgh, UK; 2 Division of Clinical Neurosciences, University of Edinburgh, Western General Hospital, Edinburgh, UK

## Background

Treating acute ischaemic stroke patients with iv-rtPA is of overall benefit. An associated increase in the risk of symptomatic intracranial haemorrhage (SICH) may cause considerable harm. We investigated (i) whether novel or existing prediction models could predict SICH or poor functional outcome in rtPA treated patients and (ii) whether ischemic stroke patients at either a high predicted risk of SICH or poor functional outcome experienced less benefit from rtPA.

## Methods

We used the IST-3 trial data, an international, multicentre, open treatment randomised trial of rtPA versus control in 3035 acute ischemic stroke patients. We developed and internally evaluated a multivariate logistic regression model for SICH following rtPA including variables identified as important in a systematic review. We compared the discrimination (area under the receiver operating characteristic curve (AUROCC)) of our model with those existing in the medical literature. We calculated the absolute risk reduction of death or dependency with rtPA in patients at a low, medium or high predicted risk of SICH or poor functional outcome with each model.

## Results

Our model had similar discrimination for SICH (AUROCC 0.68 95% CI: 0.63-0.74) to nine previously developed models (HAT, SEDAN, SITS, GRASPS, SPAN-100, Stroke-TPI, DRAGON, THRIVE and a model with NIHSS and age). There was no evidence that patients at high predicted risk of SICH or poor functional outcome after stroke derived less benefit from rtPA.

## Conclusions

We found no evidence to support a stratified approach in administering rtPA to acute ischaemic stroke patients at a high predicted risk of intracranial haemorrhage or poor functional outcome.

